# Fab N-Glycosylation in IgG: Implications in Physiological and Pathological Immune Regulation

**DOI:** 10.3390/biom15111508

**Published:** 2025-10-25

**Authors:** Shuqi Chen, Feiyuan Yu, Binliang Huang, Ganbo Liang, Jieyi Xu, Yuning Lin, Qian Xu

**Affiliations:** 1 International Science and Technology Cooperation Base for Molecular Medicine, Department of Pathology, Shantou University Medical College, Shantou 515041, China; 2 Department of Cell Biology and Genetics, Shantou University Medical College, Shantou 515041, China; 3 Department of Clinical Laboratory Medicine, Shantou University Medical College, Shantou 515041, China; 4 Shantou University Medical College, Shantou 515041, China

**Keywords:** N-glycosylation, IgG Fab fragment, autoimmune diseases, pregnancy, tumor, IgG4

## Abstract

Compared with classical Fc N-glycosylation, Fab N-glycosylation displays site heterogeneity and structural diversity. It contributes to immune regulation by modulating antibody stability, half-life, and antigen-binding activity, as well as by mediating blocking antibody effects. This review highlights the expression patterns and potential mechanisms of Fab N-glycosylated IgG in autoimmune diseases, pregnancy-induced immune tolerance, and tumor immune evasion, and discusses its structural and functional similarities to IgG4. Although Fab N-glycosylation plays an important role in both physiological and pathological conditions, the complexity of its glycan structures and variability in glycosylation sites hinder a precise understanding of its functional impacts. Clarifying these aspects is expected to emerge as a major focus in glycomics and antibody engineering research.

## 1. Introduction

**Figure 1:**
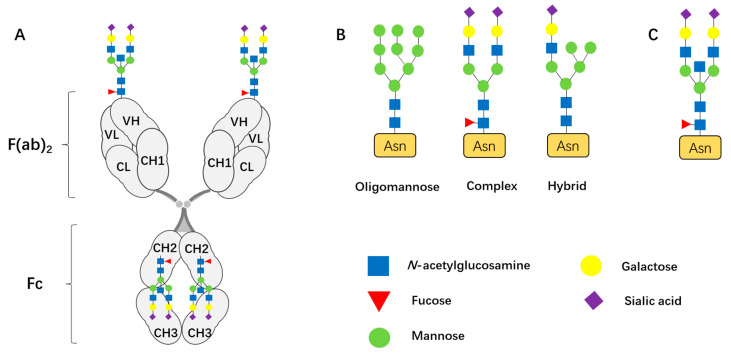
Diagram of N-Linked Glycans on IgG. (**A**) Fab N-glycosylation is typically located in the variable region, while Fc N-glycosylation is in the CH2 domain. (**B**) Three major glycan types: Oligomannose-type, Complex-type and Hybrid-type. (**C**) A bisecting GlcNAc is added to the core of hybrid or complex-type glycans, forms a new subtype of glycan termed bisecting GlcNAc.

Immunoglobulins are soluble serum glycoproteins and play crucial roles in the human immune system. The basic structure of IgG consists of two identical heavy chains and two light chains linked by disulfide bonds to form a Y-shaped monomer (Figure 1). Based on differences in the antigenicity of the heavy chains, immunoglobulins are classified into five classes: IgM, IgG, IgA, IgD, and IgE. Among these, IgG is the most abundant immunoglobulin in the blood, comprising approximately 75% of the total serum immunoglobulin. It plays an indispensable role in various infectious and immune-related diseases [1,2]. As a glycoprotein, IgG contains a conserved N-glycosylation site at position N297 in the CH2 region of its Fc fragment. The Fc N-glycan is predominantly a complex biantennary structure based on core fucose, which is essential for the antibody’s structural integrity and significantly contributes to many of the effector functions it mediates [3,4]. Research dating back to 1983 demonstrated that the glycan on the IgG Fc region can influence its functional properties, such as reducing its ability to induce antibody-dependent cell-mediated cytotoxicity (ADCC) [5]. Fc N-glycan is critical both for maintaining the structural stability and thermal resilience of the IgG Fc domain and for modulating the diversity of its effector functions [6]. Fc N-glycan interacts with different Fc receptors to regulate immune responses. For example, a lower level of fucosylation in the IgG Fc N-glycan enhances binding to FcγRIIIa by 50-fold, as well as boosting ADCC mediated by cytotoxic cells such as NK cells. Conversely, increased galactosylation and sialylation promote stronger antibody-dependent cellular phagocytosis (ADCP) by macrophages [7,8]. In individuals lacking N-glycans, IgG1 and IgG3 fail to bind to FcγRI and FcγRII, impairing the activation of immune cells by immune complexes [9]. Additionally, IgG Fc N-glycan modulates its affinity for complement binding, such that galactose-modified Fc N-glycans enhance the affinity of IgG for complement C1q, thereby promoting complement-dependent cytotoxicity (CDC) [10,11] (Table 1).

Recent studies have shown that approximately 10–25% of IgG Fab fragments in the serum of healthy individuals carry N-glycans [15,16]. However, our understanding of the role of IgG Fab N-glycans in immune regulation remains less well understoodd than that of Fc glycans. It has been observed that the N-glycans in the Fab region also adopt a biantennary structure, similar to those found in the Fc region, with fucose as the core. These glycans are branched into two arms, which may contain mannose, N-acetylglucosamine (N-GlcNAc), sialic acid, and galactose depending on the glycan type. Based on the structure of the branching ends, Fab glycans can be categorized into three major types: (1) Oligomannose-type glycans, where the branch ends consist entirely of mannose; (2) Complex-type glycan, in which at least two of the branch ends are N-acetylglucosamine residues, which can further extend to include galactose and sialic acid; (3) Hybrid-type glycan, where one branch end consists of mannose and the other consists of N-acetylglucosamine (Figure 1). Studies have revealed significant structural differences between Fab and Fc glycans. Fc glycans exhibit higher levels of fucosylation, while Fab glycans are characterized by higher levels of galactosylation and sialylation, a greater occurrence of bifurcated GlcNAc, and the presence of high-mannose structures [17,18]. Additionally, Margni et al. reported that some IgG molecules exhibit asymmetry, with one end of the Fab region carrying high-mannose glycans, while the other end does not [19]. This type of IgG, referred to as asymmetric IgG, is capable of binding to Concanavalin A (ConA). Furthermore, because the CH1 region of the heavy chain or the constant region of the light chain in the Fab fragment lacks N-linked glycosylation sites (Asn-X-Ser/Thr, where X can be any amino acid except proline), oligosaccharides in the IgG Fab region are restricted to the variable regions of the heavy or light chains, a phenomenon commonly referred to as Fab variable domain glycosylation (VDG) [20]. Notably, studies have demonstrated that, in addition to IgG with high-mannose-type Fab N-glycans, IgG with complex-type Fab N-glycans can also bind to ConA [21].

Accumulating evidence indicates that the N-glycosylation sites in the Fab region are the result of somatic mutations driven by antigen specificity, and these glycosylation sites are positively selected and are relatively infrequent in normal B cells [22]. Numerous studies have explored the effects of IgG Fab-glycans. On one hand, Fab N-glycans can influence the stability and half-life of antibodies in vivo [23,24]. On the other hand, as many Fab N-glycans are located near or within the antigen-binding site, it is believed that they can modulate antigen–antibody binding by altering antibody affinity. For instance, Wright et al. used site-directed mutagenesis to show that carbohydrates in the second complementarity-determining region (CDR2) of the heavy chain can increase antibody affinity by a factor of ten. Conversely, in some cases, the presence of Fab N-glycans may reduce or even abolish antigen binding, or have no effect [22,25,26]. The variability in these glycosylation effects is likely influenced by the position and structure of the N-glycan. Interestingly, it is currently believed that Fab N-glycosylated IgG can function as a blocking antibody [27]. This is because carbohydrates near the antigen-binding site in one of the Fab arms may block specific antigen binding, while allowing IgG to interact with other molecules without interfering with the antigen-binding site, thereby promoting antigen specificity.

## 2. Autoimmune Diseases and Fab-Glycosylated IgG

Autoimmune diseases affect approximately 5–8% of the global population [28], and increasing evidence suggests that Fab-glycosylated IgG may play a role in the onset and progression of many autoimmune disorders. In primary Sjögren’s syndrome, approximately 24% of serum IgG molecules carry N-glycans in the Fab region [29]. Anti-Desmoglein 3 (Dsg3) IgG antibodies from patients with pemphigus vulgaris (PV) exhibit significantly elevated levels of Fab glycosylation [30]. Similarly, anti-proteinase 3 (PR3) and anti-myeloperoxidase (MPO) antibodies in individuals with ANCA-associated Vasculitis (AAV) also showed increased Fab glycosylation [30,31] (Table 2).

Among these diseases, rheumatoid arthritis has been most extensively studied. Hafkenscheid et al. found that ACPA-IgG Fab regions isolated from the blood and synovial fluid of rheumatoid arthritis (RA) patients contain N-linked glycans. It is estimated that over 90% of ACPA-IgG variable regions carry Fab N-glycans, while no such phenomenon is observed in ACPA-IgM [32,33]. A study revealed that ACPA-IgG exhibits a near 100% frequency of N-glycosylation sites introduced by somatic hypermutation, far exceeding other highly mutated mAbs from RA patients and controls [34]. ACPA-IgG with highly sialylated Fab N-glycans may appear earlier during disease development, as elevated levels of ACPA-IgG Fab N-glycan can be detected in many RA patients before disease onset (Table 2). The high degree of sialylation suggests that immunoglobulin-like lectins (Siglecs) may act as potential receptors [15,35,36]. Additionally, compared to RA patients, first-degree relatives carrying ACPA-IgG show significantly lower Fab glycosylation levels. First-degree relatives who later develop RA exhibit widespread Fab glycosylation even before disease onset, indicating that ACPA-IgG Fab glycosylation levels may serve as a predictive biomarker for the development of ACPA-positive RA [37].

In myasthenia gravis (MG) patients, the frequency of MuSK-IgG and AChR-IgG Fab N-glycosylation sites is elevated in the circulatory system compared to healthy individuals, though this does not affect their binding capacity [38]. *Sambucus nigra *agglutinin (SNA) chromatography indicated elevated Fab variable domain glycosylation (VDG) levels in anti-Smith and anti-dsDNA antibodies from patients with systemic lupus erythematosus (SLE), but not in the less disease-specific anti-Ro52 antibodies [30]. In IgG4-related diseases, it has been demonstrated that IgG4 Fab N-glycosylation is increased in patient serum, primarily due to sialylation [39] (Table 2).

The increased Fab N-glycans observed in multiple autoimmune diseases may facilitate the escape of autoreactive B cells from tolerance checkpoints in the germinal center. On one hand, the addition of Fab glycosylation may alter the affinity of the B cell receptor (BCR) for its specific antigen, with this change potentially enhancing or reducing affinity depending on the nature of the antibody–antigen interaction, which warrants further investigation [35]. If antigen affinity is enhanced, it could provide a selective advantage to autoreactive B cells [33]. On the other hand, evidence suggests that Fab N-glycans may interact with lectins in the microenvironment, leading to BCR crosslinking and providing survival signals, thereby conferring a survival advantage to autoreactive B cells [40]. However, the exact role of Fab-glycosylated IgG in disease activity, severity, and prognosis in autoimmune diseases remains incompletely understood.

**Table 2:** Fab Glycosylation in Autoimmune Diseases.

Autoimmune Disease	Antibody with Fab Glycosylation	Glycosylation Type
Rheumatoid Arthritis (RA) [33]	ACPA	Complex (highly sialylated)
Systemic Lupus Erythematosus (SLE) [30]	anti-Smith anti-dsDNA	unknown
Myasthenia Gravis (MG) [38]	anti-MuSK anti-AchR	unknown
ANCA-associated Vasculitis (AAV) [30]	anti-PR3 anti-MPO	Complex
Pemphigus Vulgaris (PV) [30]	anti-Dsg3	unknown
Primary Sjögren’s Syndrome (pSS) [29]	anti-SS-A/B	Complex
Immunoglobulin G4-related disease (IgG4-RD) [39]	IgG4	Complex (Sialylated)

ACPA, anti-citrullinated protein antibody; anti-dsDNA, anti-double stranded DNA; anti-MuSK, anti-muscle-specific tyrosine kinase; anti-AchR, anti-acetylcholine receptor; anti-PR3, anti-proteinase 3; anti-MPO, anti-myeloperoxidase; anti-Dsg3, anti-desmoglein 3; anti-SS-A/B, anti-Sjögren’s syndrome-related antigen A/B.

## 3. Immune Tolerance in Pregnancy and Fab-Glycosylated IgG

During pregnancy, trophoblast cells invade the decidual layer and form a placenta containing both maternal and paternal genetic material, which poses a potential risk of immune rejection by the maternal immune system. Interestingly, during pregnancy, the maternal immune system does not mount a corresponding rejection response to the fetus, despite it being a “non-self antigen” [41,42]. This unique immune tolerance during pregnancy involves significant contributions from cells of the innate immune system, such as decidual NK cells and antigen-presenting cells, as well as the balance of Treg cells and Th1-Th2 cytokines [43,44,45,46,47]. Furthermore, B cells and their products likely play an important role in promoting immune tolerance during pregnancy. B cells can contribute by secreting immunoregulatory cytokines (such as IL-10) and asymmetric IgG, a kind of Fab-glycosylated IgG, thereby promoting pregnancy immune tolerance [48].

Studies have shown that during normal pregnancy, the percentage of Fab-glycosylated IgG in the serum increases, reaching a peak of 50% in the second month of pregnancy. 80% of the IgG molecules bound to normal full-term placental tissue exhibit anti-paternal activity, suggesting that Fab-glycosylated IgG may serve as a mediator of protective immune responses during pregnancy [49,50,51]. A study revealed that Fab-glycosylated IgG levels were approximately 20% lower in newborns than in their mothers, indicating that Fab-glycans may reduce IgG-FcRn binding, which negatively affects the transplacental transfer of Fab-glycosylated IgG [52].

**Figure 2:**
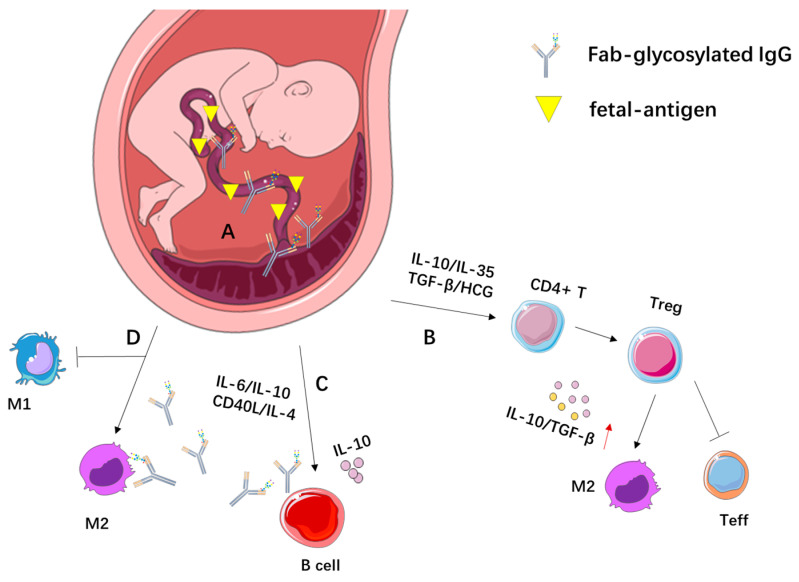
Potential Mechanisms of Fab-Glycosylated IgG in Pregnancy. (**A**) Fab-glycosylated IgG does not bind to fetal-derived antigens, thereby avoiding the initiation of downstream immune responses and reducing maternal rejection of the fetus. (**B**) Trophoblast cells secrete cytokines that promote the differentiation of T cells into regulatory T cells (Tregs). (**C**) Stimulation of term maternal-fetal interface B cells with a combination of CD40L, IL-6, IL-10, and IL-4 promotes increased production of Fab-glycosylated IgG. (**D**) Trophoblast cells secrete various cytokines that promote macrophage polarization toward the M2 phenotype. Additionally, the mannose residues on Fab-glycosylated IgG can bind to mannose receptors on macrophages, thereby modulating downstream immune responses.

The production of substantial amounts of Fab-glycosylated IgG during pregnancy may be promoted by cytokines such as IL-6, secreted by trophoblast and other cells in the pregnancy environment, which regulates the activity of UDP-Glc (Uridine Diphosphate Glucose) glycoprotein glucosyltransferase in B lymphocytes. This, in turn, promotes changes in IgG heavy chain folding, exposing new sequences that facilitate glycosylation. Canellada et al. found that mixing CD40L with IL-6, IL-10, and IL-4 to stimulate B cells isolated from the maternal-fetal interface of full-term pregnancy significantly induced the production of Fab-glycosylated IgG [27,53,54,55,56] (Figure 2). However, Gu et al. discovered that in B lymphocyte-deficient mice, maternal mice could not produce IgG, but trophoblast cells in the placenta still contained IgG. This indicates that the IgG in the placental microenvironment during pregnancy is not derived from B cells but from maternal trophoblast and endothelial cells. A significant portion of this IgG is abnormally glycosylated at the Fab arms, forming Fab-glycosylated IgG [57,58]. Regardless of the cell type responsible for the production of Fab-glycosylated IgG, its structural characteristics suggest that the asymmetry of the single Fab region containing oligomannose structures may block the binding of placental antigens to the maternal immune system, thereby halting various downstream immune responses and protecting the fetus from maternal immune attack. In fact, a low percentage of asymmetric IgG in the maternal serum during the first trimester of pregnancy may correlate with poor pregnancy outcome [55]. Additionally, studies have found that prior injection of particulate antigens in rats before mating preferentially induces the synthesis of anti-paternal IgG asymmetric antibodies. As a result, in women with recurrent spontaneous miscarriages, injection of paternal lymphocytes has shown some therapeutic benefit [59]. These findings provide evidence suggesting that the increased concentration of Fab-glycosylated IgG during pregnancy may play a role in establishing the immune tolerance environment in the placental microenvironment.

## 4. Fab-Glycosylated IgG in Tumor Immune Escape and Antibody Therapy

In 2002, Schreiber et al. proposed the theory of tumor immune editing, dividing it into three phases: immune elimination, immune equilibrium, and immune escape [60]. Tumor immune escape is a key step in malignant progression and one of the main obstacles to immunotherapy. The mechanisms of immune escape are generally considered to include: (1) loss of expression of tumor-specific antigens; (2) induction of immune cell dysfunction or apoptosis through the expression of immune checkpoint molecules (such as PD-L1); and (3) the formation of an immunosuppressive microenvironment in the tumor-infiltrating region [61].

**Figure 3:**
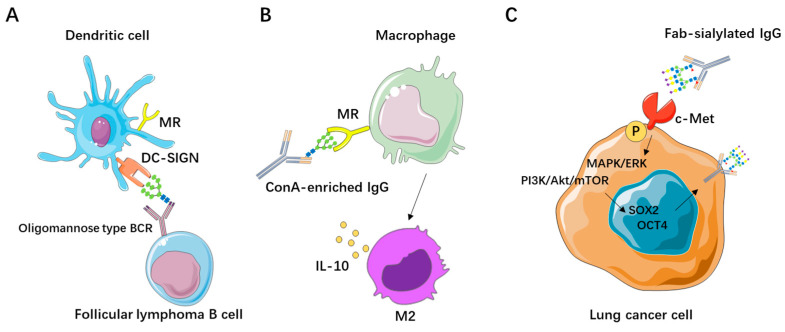
Effect of Fab glycosylation in tumors. (**A**) Follicular lymphomas may interact with MR, DC-SIGN, via high-mannose glycans, presumably enhancing tumor survival. (**B**) ConA-enriched IgG binds to the mannose receptor (MR) on macrophages, promoting macrophage M2 polarization and facilitating tumor immune escape. (**C**) Cancer-derived IgG, which exhibits Fab-region sialylation, promotes lung cancer cell stemness by activating the c-Met/Akt/Erk signaling axis, further stimulates SOX2, and thereby forms a self-perpetuating Fab-sialylated IgG/c-Met/SOX2/Fab-sialylated IgG signaling loop.

Numerous studies have shown that Fab-glycosylated IgG plays a critical role in certain hematologic malignancies and may contribute to immune escape, which could promote tumor initiation and progression. In follicular lymphoma (FL), immunoglobulins expressing genes with somatic mutations are commonly found; these mutations are present in nearly all diagnosed FL cases and are acquired early during lymphoma development. These genes are closely associated with the introduction of N-glycosylation motifs, and although the N-glycosylation sites are present in FL, they are rarely found in normal B cells, suggesting their involvement in the pathogenesis of the tumor [62,63]. Studies analyzing the glycosylation patterns of the Fab variable regions of IgG and IgM in the blood of lymphoma patients have found that most of these glycosylation sites are predominantly oligomannose structures located at antigen-binding sites, implying that they may block traditional antigen binding [12,64]. Alternatively, the presence of oligomannose may indicate that surface immunoglobulins in FL can activate malignant cells in the absence of antigen, thereby promoting tumor progression. The prevailing view is that the presence of Fab N-glycans may interact with lectins in the microenvironment, including dendritic cell-specific intercellular adhesion molecule-Grabbing Nonintegrin (DC-SIGN), or with lectins from opportunistic bacteria. Such interactions may stimulate B cell receptors (BCRs) via exposed Fab variable domains with oligomannose structures, providing additional growth and survival signals to lymphoma cells [26,40,65] (Figure 3A). Therefore, Fab-glycosylated IgG may serve as a potential therapeutic target, with BCR signaling inhibitors emerging as effective treatments for lectin-driven malignancies.

Research on Fab-glycosylated IgG in solid tumors is more limited. Klaamas et al. developed an enzyme-linked immunosorbent assay (ELISA) based on the lectin ConA to detect IgG in serum that can be purified by ConA. They found that the level of ConA-enriched IgG in the serum of gastric cancer patients was significantly higher compared to controls, and patients with lower levels of ConA-enriched IgG were associated with better survival outcomes [66]. Some researchers suggest that ConA-enriched IgG is involved in tumor progression through the N-glycans in Fc region [67,68]. However, Huang et al. discovered that Fc N-glycans in their native folded state cannot interact with the ConA affinity chromatography column, indicating that the ConA-enriched IgG promotes tumor progression through N-glycans in Fab region rather than the Fc region [69]. Furthermore, Xu et al. found that administering ConA-enriched IgG in a mouse breast cancer model promoted tumor growth and metastasis. They hypothesized that ConA-enriched IgG may facilitate tumor cell immune escape by influencing antigen–antibody binding and macrophage M2 polarization [70] (Figure 3B). In addition to oligomannose-type glycans, sialylation in the Fab region is also closely associated with tumors. Huang et al. found that cancer-derived IgG exhibits sialylation modification in its Fab region, which can promote lung cancer cell stemness by activating the c-Met/Akt/Erk signaling axis. It further stimulates SOX2, thereby forming a self-perpetuating signaling loop of Fab-sialylated IgG/c-Met/SOX2/Fab-sialylated IgG [71] (Figure 3C).

Fab-glycosylated IgG also demonstrates versatility in tumor therapy. Adalimumab, a fully humanized anti-TNF-α IgG1 monoclonal antibody, has been shown to have significantly enhanced conformational stability when N-glycans are added to its Fab domain, thereby increasing its steric hindrance and preventing aggregation, a major challenge in biopharmaceutical production [72,73]. Additionally, cetuximab undergoes glycosylation at the Fab and Fc domains at N88 and N297 of the heavy chain, respectively. The presence of additional glycosylation sites in the variable region of the molecule results in significant charge variation and glycan heterogeneity [74,75,76]. Studies have shown that the heterogeneous and immunogenic Fab N-glycans in cetuximab’s homogeneous glycoform can be replaced with a single sialylated N-glycan, which increases its affinity for FcγRIIIa and significantly enhances ADCC activity [77]. In fact, because Fab N-glycans are highly flexible, they may cause antigen-binding epitopes targeted by drug-resistant antibodies to become more variable, altering the recognition of these drug-resistant antibodies. This suggests that the polysaccharides in the IgG Fab domain could be a useful tool in the development of therapeutic antibodies.

## 5. Association of Fab-Glycosylated IgG with IgG4

IgG exists in four subclasses in healthy humans, with their serum proportions being IgG1 (65%), IgG2 (25%), IgG3 (6%), and IgG4 (4%) [78,79]. As the least abundant and the last discovered IgG subclass, IgG4 is often regarded as a blocking antibody. Compared to the other three IgG subclasses, IgG4 has a shorter hinge region, poorer Fab extension, and unique structural characteristics in the CH2 domain, which may partially block the binding sites for FcγR and C1q in the Fc region. This can affect the mediation of downstream classical immune responses (such as ADCC, ADCP, and CDC) [80,81]. On the other hand, the cysteine-proline-serine-cysteine core sequence in the hinge region of IgG4 can produce bispecific antibodies through a Fab arm exchange (FAE) mechanism, and in certain conditions, the antibody may also be monovalent. This results in IgG4 antibodies exhibiting random specificity, which may prevent the formation of effective immune complexes with antigens. Additionally, IgG4 molecules can interact with each other via Fc-Fc binding, leading to IgG4 deposition. These structural features of IgG4 impair the body’s ability to generate normal immune responses [82,83,84].

Although IgG4 comprises about 4% of total IgG in normal human serum, studies have shown elevated levels of IgG4 in chronic inflammatory diseases such as rheumatoid arthritis [85], IgG4-related disease [86], and pemphigus vulgaris [87]. Follow-up studies revealed that some patients developed malignant tumors, suggesting a close association between IgG4 and the development of chronic inflammation and malignant tumors [80,88]. In tumor-related research, multiple teams have identified IgG4 and IgG4-positive plasma cell infiltration in tumor tissues of melanoma [89], colorectal cancer [90], extrahepatic cholangiocarcinoma [91], gastric cancer [88] and esophageal cancer [92]. These studies found that the high concentration of IgG4 correlates with poor prognosis. High-affinity tumor-specific IgG4 can competitively bind tumor-specific antigens with IgG1, thereby blocking the downstream immune clearance reactions mediated by IgG1 [80,93]. Additionally, Wang et al. discovered that IgG4 in tumor patients is nonspecific to the tumor and cannot recognize tumor tissues. However, it can bind to IgG1 that is already bound to tumor-associated antigens, thus blocking IgG1-mediated immune responses [92]. On the other hand, some tumor cells produce IL-10, which promotes plasma cells to generate IgG4, enhances the function of Treg cells, induces a Th2-type immune environment, and helps tumor immune evasion [94]. Furthermore, crosslinking of IgG4 with FcRIIb can redirect allergic M2a macrophages to the immune-suppressive M2b phenotype, inducing immune tolerance [95].

The low Fc effector function of IgG4 (which barely mediates ADCC, CDC, or ADCP) gives it a unique advantage in tumor immunotherapy scenarios that focus on blocking target signaling (such as the PD-1/PD-L1 pathway) without relying on immune cell killing [96]. As a member of the immunoglobulin superfamily, the extracellular IgV domain of PD-1 contains several conserved N-glycosylation sites (such as N49, N58, N74, and N116). These sites are typically modified with N-glycans featuring a bi-antennary structure as the core backbone. Among them, the glycan at the N58 site directly participates in the binding of camrelizumab, which serves as a structural basis for the mechanism of action of this antibody [97]. To prevent FAE from affecting the activity of IgG4 subclass antibodies, a mutation was introduced in the hinge region at position 228 (serine to proline) to stabilize the structure [98]. Unfortunately, not all patients respond to immune checkpoint therapies, and some may even experience tumor progression (Hyperprogressive Disease, HPD) during treatment [99,100,101]. The facilitation of IgG4 heavy chain dissociation and Fc-Fc-mediated local immunosuppression by glutathione has been proposed as a mechanistic explanation for tumor hyperprogression [102].

As mentioned earlier, Fab-glycosylated IgG is also considered a blocking antibody. Research has shown that different immunoglobulin subclasses exhibit distinct Fab glycosylation patterns, with IgE and IgG4 carrying more Fab glycans compared to other immunoglobulins [103,104,105]. In terms of function, Fab-glycosylated IgG shares several features with IgG4. First, although Fab-glycosylated IgG has two antigen-binding sites, the Fab glycans can affect antigen affinity [106]. IgG4, which produces bispecific antibodies through FAE, cannot crosslink with antigens, and both fail to form effective immune complexes [1,107]. Second, both exhibit competitive binding characteristics. Fab-glycosylated IgG competes with non-glycosylated IgG for the same antigen, while IgG4 competes with IgG1 for the same antigen, both ultimately leading to an inability to effectively initiate downstream classical immune responses [89,92]. Additionally, both can promote macrophage polarization toward the M2 phenotype, enhancing immune tolerance [70,90]. Lastly, elevated levels of both IgG4 and Fab-glycosylated IgG have been observed in tumor progression, autoimmune diseases, and chronic bacterial infections [35,80,88]. These findings suggest that there may be important connections between Fab-glycosylated IgG and IgG4 in the body, and analyzing both together could provide valuable insights into immune evasion mechanisms (Table 3).

## 6. Conclusions

In summary, Fab-glycosylated IgG plays a significant role in immune regulation under both physiological and pathological conditions. It may be involved in mechanisms such as immune tolerance during pregnancy and immune evasion in tumors, and shares many structural and functional similarities with IgG4. However, due to the complexity of N-glycan structures, research in this area remains limited. For example, the N-glycosylation sites on the IgG Fab region are not fixed, with varying specific sites and complex glycan structures, and the precise impact of these variations on biological function remains unclear. Further research into the various roles and underlying mechanisms of Fab-glycosylated IgG in immune processes is warranted. This could provide deeper insights into the regulation of humoral immune responses, particularly in immune evasion, and could inform the development of antibody-based therapies.

## Figures and Tables

**Table 1:** Monosaccharides in N-Linked Glycans: Roles and Applications.

Monosaccharide	Primary Role in N-Glycan Structure	Applications & Significance
*N*-Acetylglucosamine (GlcNAc)	Forms the core pentasaccharide (Man3GlcNAc2) of N-glycan.	Critical for the stability, solubility, and half-life [3]
Mannose (Man)	Forms the core pentasaccharide (Man3GlcNAc2) of N-glycan.	- High-mannose glycans on IgG are increased in malignant tumors [12] - Macrophages and dendritic cells express mannose receptor that bind to mannose residues, triggering downstream immune effects [13]
Galactose (Gal)	- Often added as the final sugar to the end of a glycan chain. - Provides the attachment point for sialic acid.	- Plays a role in inflammatory responses by mediating cell–cell interactions. - The galactose on IgG antibodies influences their ability to activate the complement system [10]
Fucose (Fuc)	Core fucosylation	Core fucosylation on IgG drastically reduces their ADCC activity [5]
Sialic acid (Sia)	Terminal modification	Highly sialylated IgG enhances anti-inflammatory activity by modulating macrophage and dendritic cell functions [14]

**Table 3:** A Comparison of Fab-glycosylated IgG and IgG4.

Feature	Fab-Glycosylated IgG	IgG4
**Glycosylation Site**	Fab and Fc (Asn-297)	Fab (pathological condition) and Fc (Asn-297)
**Characteristics**	- Diverse glycoforms: oligomannose-type, complex-type, or hybrid-type glycans - Glycosylation levels may exhibit asymmetry between the two Fab regions	- Weaker binding affinity to Fcγ receptors (except FcγRI) and C1q - Fab-arm exchange
**Functional Impact**	Modulates antigen binding; enhances BCR signaling; interacts with lectins to transduce signals and modulates the immune microenvironment [106].	- Minimally induces NK cell-mediated ADCC and complement-dependent CDC effects [80] - Fab-arm exchange can inhibit immune complex formation and neutralize antigen [82]
**Association with Autoimmune Diseases**	Strongly correlated with specific autoantibodies, such as ACPA in RA [33].	IgG4-related disease is characterized by massive infiltration of IgG4-positive plasma cells and fibrosis [86].
**Association with Cancer**	- In B-cell malignancies, the variable regions of tumor BCR frequently carry oligomannose-type glycans [12] - Fab glycans are found closely associated with the progression of some solid tumors [66,70]	- The weak effector functions of IgG4 make it an ideal choice for certain cancer immune checkpoint inhibitors (e.g., Nivolumab, Pembrolizumab) [108] - Elevated levels of IgG4 have been reported in patients with cancer [88,92]
**Summary**	Fab glycosylation is an acquired modification. It participates in disease development and serves as a potential biomarker for diseases.	IgG4 is a natural subclass of IgG, characterized by structural features that determine its inherent functional properties (weak effector functions and anti-inflammatory activity).

## Data Availability

No new data were created or analyzed in this study.

## Abbreviations

The following abbreviations are used in this manuscript:

ADCC	Antibody-Dependent Cell-mediated Cytotoxicity
ADCP	Antibody-Dependent Cellular Phagocytosis
CDC	Complement-Dependent Cytotoxicity
N-GlcNAc	N-acetylglucosamine
ConA	Concanavalin A
CDR2	the Second Complementarity-Determining Region
PSS	Primary Sjögren’s Syndrome,
Dsg3	Desmoglein 3
PV	Pemphigus Vulgaris
PR3	Proteinase 3
MPO	Myeloperoxidase
ANCA	Anti-Neutrophil Cytoplasmic Antibody
AAV	ANCA-associated Vasculitis
ACPA	Anti-Citrullinated Protein Antibody
RA	Rheumatoid Arthritis
MG	Myasthenia Gravis
MuSK	Muscle-Specific Tyrosine Kinase
AChR	Acetylcholine Receptor
SNA	*Sambucus Nigra Agglutinin*
VDG	Variable Domain Glycosylation
SLE	Systemic Lupus Erythematosus
BCR	B Cell Receptor
UDP-Glc	Uridine Diphosphate Glucose
FL	Follicular Lymphoma
DC-SIGN	Dendritic Cell-Specific Intercellular adhesion molecule-Grabbing Nonintegrin
ELISA	Enzyme-Linked Immunosorbent Assay
PD-1	Programmed Cell Death Protein 1
FAE	Fab Arm Exchange
PD-L1	Programmed Cell Death Ligand-1
HPD	Hyperprogressive Disease
